# Evaluating epistatic interaction signals in complex traits using quantitative traits

**DOI:** 10.1186/1753-6561-3-s7-s82

**Published:** 2009-12-15

**Authors:** Odity Mukherjee, Krishna Rao Sanapala, Padmanabhan Anbazhagana, Saurabh Ghosh

**Affiliations:** 1 National Center for Biological Sciences, Bangalore, India; 2 Indian Statistical Institute, Kolkata, India; 3 National Institute of Mental Health and Neurosciences, Bangalore, India

## Abstract

Rheumatoid arthritis (RA) is a complex, chronic inflammatory disease implicated to have several plausible candidate loci; however, these may not account for all the genetic variations underlying RA. Common disorders are hypothesized to be highly complex with interaction among genes and other risk factors playing a major role in the disease process. This complexity is further magnified because such interactions may be with or without a strong independent effect and are thus difficult to detect using traditional statistical methodologies. The main challenge to analyze such gene × gene and gene × environment interaction is attributed to a phenomenon referred to as the "curse of dimensionality." Several combinatorial methodologies have been proposed to tackle this analytical challenge. Because quantitative traits underlie complex phenotypes and contain more information on the trait variation within genotypes than qualitative dichotomy, analyzing quantitative traits correlated with the affection status is a more powerful tool for mapping such trait genes. Recently, a generalized multifactor dimensionality reduction method was proposed that allows for adjustment for discrete and quantitative traits and can be used to analyze qualitative and quantitative phenotypes in a population based study design.

In this report, we evaluate the efficiency of the generalized multifactor dimensionality reduction statistical suite to decipher small interacting factors that contribute to RA disease pathogenesis.

## Introduction

Rheumatoid arthritis (RA) is a complex chronic inflammatory disease implicated to have several plausible candidate loci. Many genetic studies have been undertaken and only two genes, *HLA-DRB1 *and *PTPN22*, have been reported to be associated with disease [1-4]. Although these findings are encouraging, they may not account for all the genetic variations in RA because no direct pathogenic role of these molecules have been established in the development of the disease pathogenesis. Common disorders like RA are hypothesized to be highly complex, with interaction among genes and other risk factors playing a major role in the disease process. This complexity is further magnified because such interactions may be with or without strong independent main effect, and thus difficult to detect using traditional statistical methodologies [5]. The main challenge to analyzing epistatic interactions is attributed to a phenomenon referred to as the "curse of dimensionality," which is a problem caused by the exponential increase in volume associated with adding extra dimensions to a mathematical space. Thus, while analyzing interactions among several loci for a complex phenotype, contingency tables in higher dimensions suffer from the problem of sparse data, leading to unreliable risk estimates. Several combinatorial methodologies have been proposed to overcome this analytical challenge: multifactor dimensionality reduction (MDR) [6]; combinatorial partitioning method (CPM) [7] and restricted partition method (RPM) [8]. Although these methods have been used by several research groups, there exist some limitations in their current form: a) inability to adjust for covariates' MDR, b) inability to use quantitative phenotypes, and c) computationally intense algorithms.

Thus, there is a need to develop and evaluate more powerful statistical methodology so as to decipher small interacting factors that contribute to disease pathogenesis. Because quantitative traits underlie complex phenotypes and contain more information on the trait variation within genotypes than qualitative dichotomy, analyzing quantitative traits correlated with the affection status is a more powerful tool for mapping complex trait genes. Recently, a generalized MDR (GMDR) method was proposed that allows for adjustment for discrete and quantitative traits and can be used to analyze qualitative and quantative phenotypes in a population based study design [9].

In this report, we use the GMDR statistical suit to evaluate its efficiency to decipher small interacting factors that contribute to RA disease pathogenesis, using the two quantitative traits [anti-CCP (anti-cyclic citrullinated peptide) and RFUW (rheumatoid factor)] as covariates for classifying the data into high and low risk groups.

### Data analysis

**Figure 1:**
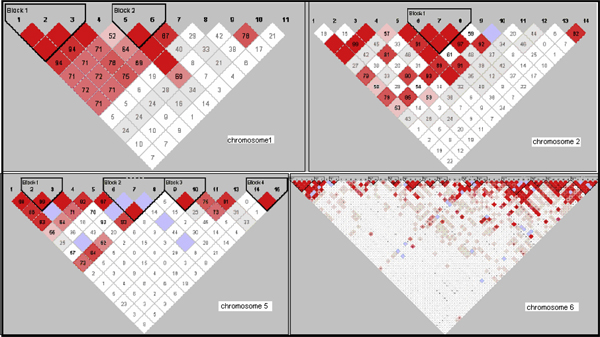
**LD block structure across the chromosomal regions used in this study**. The figures show the output of Haploview (version 3.32) LD Plot where each square (with D' values written within the box) represents a pair-wise LD relationship between the two SNPs. Red squares indicate statistically significant LD between the pair of SNPs as measured by the D' statistic. Darker colors of red indicate higher values of D', up to a maximum of 1. White squares indicate pair-wise D' values <1 with no statistically significant evidence of LD.

An initial screen of data for quality control was performed for the markers selected for the current study. Hardy-Weinberg equilibrium (HWE) was estimated in the case, control, and combined groups using the Haploview program (version 3.32). To understand the degree of correlation between the SNPs, linkage disequilibrium (LD) was estimated using the Haploview program (version 3.32). The D' statistics for the same is presented in Figure 1. Such information is essential when analyzing data employing cross-validation steps because it is possible that the algorithm might identify different SNPs (but in tight LD) for each of its cross-validation intervals [2]. This was followed by the GMDR analysis for detecting epistatic interactions.

## Methods

### Sample and marker selection

In the current study, we used the Genetic Analysis Workshop 16 (GAW16) RA case-control data set (Problem 1) comprising a total of 2062 sample (case = 868, control = 1194), typed on the 550 k Illumina chip. To evaluate the efficiency of the GMDR algorithm to detect small epistatic interactions involved in RA pathogenesis, analysis was performed on chromosomes 1, 2, 5, and 6, which have shown strong positive association earlier with the phenotype [1-4,10]. Because quantitative trait information was available for only cases, interaction analysis using GMDR was performed on the RA cases (*n *= 867).

### Interaction studies

The GMDR is a score-based algorithm based on the MDR framework. Briefly, the MDR uses a novel constructive induction algorithm to facilitate the detection of nonlinear interactions among multiple discrete genetic and/or environmental factors that are predictive of a discrete clinical endpoint [11]. Multi-locus genotype combinations are classified as high-risk or low-risk genotype combinations using a threshold that is equal to the ratio of cases and controls. The best model is selected as the combination of marker with maximum cross-validation consistency and minimum prediction error. GMDR works on the same framework of MDR, but is a score-based algorithm. Improving on the original MDR, it can be used on both qualitative and quantitative traits, it allows adjustment for covariates and better handles unbalanced population based data. For the current study, we employed two methods to compute the scores (described below) for GMDR using the QTL information provided in the data set:

1. The GMDR scoring method: The GMDR method uses the original MDR data reduction method, with the ratio of cases to control being replaced by scores in each cell to discriminate between high risk and low risk followed by determining classification accuracy and prediction error. A detailed description of the methodology can be found elsewhere [9]. This generalization of the original MDR algorithm a) allows increased flexibility to use covariates, b) is able to handle both dichotomous and continuous phenotypes, c) can be applied to a variety of population-based study designs (e.g., unbalanced case control samples.)

2. We formulated a detailed scoring methodology by using the expression *S *= exp(*y*)/1+exp(*y*), where *y *is the standardized quantitative trait. In brief, this was done by computing the mean and standard deviation (SD) of the quantitative trait. Scores where then assigned by subtracting the mean from the individual's quantitative trait value and then dividing it by the SD.

## Results

### Marker selection

In the current study we used GMDR algorithm to evaluate its efficiency in detecting gene-gene interactions in the complex RA phenotype. For this we used markers information from the GAW16 data set from regions that have been previously implicated in RA. Additional file 1 lists the markers and their chromosomal position used in this analysis. All the markers selected were in HWE (data not shown). None of the regions selected showed extensive LD between the markers (Figure 1).

### Interaction studies

While the MDR software is designed to classify individuals into high risk and low risk groups, GMDR is a score-based method in which the ratio of cases to control is replaced by scores in each cell to discriminate between high risk and low risk cells and then assessing classification accuracy and prediction error. GMDR was performed on the genotype data (cases only) from the GAW16 Problem 1 data set with the computed scores. The phenotype scores used in the analysis were generated using the built-in GMDR scoring method and a detailed scoring method (described above in the Methods section). Analysis was performed individually for the separate chromosomal regions. An exhaustive search was performed to identify all possible one- to five-locus models. We report the prediction accuracy and cross-validation consistency for the most significant models identified by GMDR, the results of which are summarized in Table 1. GMDR was able to identify small interacting factors in the regions analyzed in this study. This substantiates the efficiency of the GMDR and the candidate loci for harboring disease-associated markers.

**Table 1:** Summary of the best models obtained using GMDR algorithm for the quantitative trait RFUW (IgM)^a^

	No. loci			
	
	1	2	3	4
SNPs in best model	rs2156875	rs1517352	rs11203368	rs3024912
		rs3024896	rs6683201	rs1517352
			rs3789607	rs4555370
				rs231726
Chromosome	6	6	1	2
Gene	*HLA-B*	*CTLA4*	*PADI4, PTPN22*	*STAT4, CTLA4*
Predictive accuracy	0.5739	0.5577	0.5069	0.5396
Cross-validation consistency	10	6	5	7
Sign test *p*-value	0.017	0.001	0.377	0.0547

^a^All models used the GDMR scoring method.

## Discussion

Rheumatoid factor (RFUW) has been widely used as a screening test for patients with RA. RFUW is prognostically useful because it correlates with functional and radiographic outcomes in RA [12]. More recently, the anti-cyclic citrullinated peptide (anti-CCP) antibody has been developed, with a sensitivity of ~68% and specificity of 97% [13,14]. Together, these clinical values serve as important indicators of the disease status and are routinely used in clinical setting to aid in diagnosis. Common disorders like RA are hypothesized to be highly complex, with interaction among genes and other risk factors playing a major role in the disease process. Powerful statistical methodology has been developed to overcome these challenges to decipher small epistatic interactions that are characteristic of such phenotypes. Because quantitative traits underlie complex phenotypes and contain more information on the trait variation within genotypes than qualitative dichotomy, we used the anti-CCP value and the RFUW values provided in the GAW16 Problem 1 data set to evaluate the recently developed GMDR algorithm to detect small interacting markers for RA disease status.

In this study we used the GMDR methodology to evaluate its efficiency to detect gene-gene interactions in putative regions for RA using the anti-CCP and RFUW (IgM) values as covariates. Three out of the four models predicted reached statistical significance (Table 1). None of the high-order interactions were between correlated markers, suggesting that there might be more than one signal in these genes. For this study we had used both the anti-CCP and the RFUW values to generate scores for the GMDR analysis. Scoring based on anti-CCP value did not result in significant interaction models. Our results show that RFUW values are better predictor of high-risk and low-risk classes and further strengthen the role of RFUW (IgM) antibody as a strong prognostic factor. Detailed biological characterization of this quantitative trait are warranted.

## List of abbreviations used

anti-CCP: Anti-cyclic citrullinated peptide; CPM: Combinatorial partitioning method; GAW16: Genetic Analysis Workshop 16; GMDR: Generalized multifactor dimensionality reduction; HWE: Hardy-Weinberg equilibrium; IgM: Immunoglobulin M; LD: Linkage disequilibrium; MDR: Multifactor dimensionality reduction; RA: Rheumatoid arthritis; RFUW: Rheumatoid factor; RPM: Restricted partition method; SD: Standard deviation; SNP: Single-nucleotide polymorphism.

## Competing interests

The authors declare that they have no competing interests.

## Authors' contributions

OM carried out all statistical analysis, data interpretation, and drafted the manuscript. KRS and PA contributed in data cleaning and analysis. SG contributed in data analysis manuscript writing. All authors have read the paper and approve its contents.

## Supplementary Material

Additional file 1 SNPs used in this study. Click here for file
