# Supplementary material for: Evaluating epistatic interaction signals in complex traits using quantitative traits
Source: BMC Proc. 2009 Dec 15;3(Suppl 7):S82. doi: 10.1186/1753-6561-3-s7-s82 (PMC2795985; doi:10.1186/1753-6561-3-s7-s82)
Supplement: Additional file 1 — SNPs used in this study. [file 1753-6561-3-S7-S82-S1.pdf]

**Supplemental File 1 - SNPs used in this study**

| Chromosome | Marker[a] | SNP no. | Position | Gene |
|---|---|---|---|---|
| 1 | 1 | rs6586516 | 17498118 | *PADI4* |
| 1 | 2 | rs2477142 | 17501591 | *PADI4* |
| 1 | 3 | rs2501787 | 17503314 | *PADI4* |
| 1 | 4 | rs1886302 | 17507983 | *PADI4* |
| 1 | 5 | rs2147333 | 17519113 | *PADI4* |
| 1 | 6 | rs1635586 | 17537357 | *PADI4* |
| 1 | 7 | rs11203368 | 17539095 | *PADI4* |
| 1 | 8 | rs2240337 | 17546809 | *PADI4* |
| 1 | 9 | rs6683201 | 17553864 | *PADI4* |
| 1 | 10 | rs2428735 | 17557761 | *PADI4* |
| 1 | 11 | rs3789607 | 114167957 | *PTPN22* |
| 2 | 1 | rs6740131 | 191597827 | *STAT4* |
| 2 | 2 | rs3024912 | 191601332 | *STAT4* |
| 2 | 3 | rs3024908 | 191602386 | *STAT4* |
| 2 | 4 | rs3024896 | 191604961 | *STAT4* |
| 2 | 5 | rs16833215 | 191622044 | *STAT4* |
| 2 | 6 | rs3771327 | 191629016 | *STAT4* |
| 2 | 7 | rs1517352 | 191639709 | *STAT4* |
| 2 | 8 | rs1400656 | 191643278 | *STAT4* |
| 2 | 9 | rs2459611 | 191647432 | *STAT4* |
| 2 | 10 | rs16833239 | 191648505 | *STAT4* |
| 2 | 11 | rs4555370 | 191699704 | *STAT4* |
| 2 | 12 | rs7572482 | 191723317 | *STAT4* |
| 2 | 13 | rs926169 | 204430997 | *CTLA4* |
| 2 | 14 | rs231726 | 204449111 | *CTLA4* |
| 5 | 1 | rs2277025 | 156392673 | *HAVCR1* |
| 5 | 2 | rs10038271 | 156402979 | *HAVCR1* |
| 5 | 3 | rs7706174 | 156407976 | *HAVCR1* |
| 5 | 4 | rs17573010 | 156410409 | *HAVCR1* |
| 5 | 5 | rs4704843 | 156423731 | *HAVCR1* |
| 5 | 6 | rs12152865 | 156436345 | *HAVCR2* |
| 5 | 7 | rs13176785 | 156448854 | *HAVCR2* |
| 5 | 8 | rs919747 | 156463908 | *HAVCR2* |
| 5 | 9 | rs162889 | 131652285 | *SLC22A4* |
| 5 | 10 | rs2662314 | 131653068 | *SLC22A4* |
| 5 | 11 | rs270613 | 131668482 | *SLC22A4* |
| 5 | 12 | rs272893 | 131690961 | *SLC22A4* |
| 5 | 13 | rs2073506 | 131422637 | *IL3* |
| 5 | 14 | rs2243288 | 132045843 | *IL4* |
| 5 | 15 | rs2243290 | 132046068 | *IL4* |
| 6 | 1 | rs2517912 | 219791923 | *HLA-F* |
| 6 | 2 | rs9258170 | 29797569 | *HLA-F* |
| 6 | 3 | rs9258186 | 29800313 | *HLA-F* |

| | | | | |
|---|---|---|---|---|
| 6 | 4 | rs2523404 | 29803692 | *HLA-F* |
| 6 | 5 | rs2272874 | 29804224 | *HLA-F* |
| 6 | 6 | rs1611350 | 29806800 | *HLA-F* |
| 6 | 7 | rs1610601 | 29808162 | *HLA-F* |
| 6 | 8 | rs7741100 | 29876102 | |
| 6 | 9 | rs2523790 | 29911629 | *HLA-G* |
| 6 | 10 | rs2735007 | 29916178 | *HLA-G* |
| 6 | 11 | rs762324 | 30563865 | *HLA-E* |
| 6 | 12 | rs9264508 | 31341193 | *HLA-C* |
| 6 | 13 | rs2524099 | 31344030 | *HLA-C* |
| 6 | 14 | rs9394047 | 31344229 | *HLA-C* |
| 6 | 15 | rs2249742 | 31348700 | *HLA-C* |
| 6 | 16 | rs6906846 | 31353715 | *HLA-C* |
| 6 | 17 | rs7453967 | 31422222 | *HLA-B* |
| 6 | 18 | rs2156875 | 31425326 | *HLA-B* |
| 6 | 19 | rs2395175 | 32513004 | *HLA-DRA* |
| 6 | 20 | rs3129871 | 32514320 | *HLA-DRA* |
| 6 | 21 | rs2239804 | 32519501 | *HLA-DRA* |
| 6 | 22 | rs3129890 | 32522251 | *HLA-DRA* |
| 6 | 23 | rs13199787 | 32813254 | *HLA-DQA2* |
| 6 | 24 | rs10807113 | 32830164 | *HLA-DQA2* |
| 6 | 25 | rs7774954 | 32832167 | *HLA-DQA2* |
| 6 | 26 | rs2856997 | 32889754 | *HLA-DOB* |
| 6 | 27 | rs7383287 | 32891064 | *HLA-DOB* |
| 6 | 28 | rs2071472 | 32892598 | *HLA-DOB* |
| 6 | 29 | rs2857107 | 32893493 | *HLA-DOB* |
| 6 | 30 | rs9501239 | 33003558 | *HLA-DMB* |
| 6 | 31 | rs154978 | 33007274 | *HLA-DMB* |
| 6 | 32 | rs3132131 | 33007463 | *HLA-DMB* |
| 6 | 33 | rs154972 | 33008629 | *HLA-DMB* |
| 6 | 34 | rs181997 | 33008696 | *HLA-DMB* |
| 6 | 35 | rs3132132 | 33009912 | *HLA-DMB* |
| 6 | 36 | rs68600 | 33011702 | *HLA-DMB* |
| 6 | 37 | rs151719 | 33011878 | *HLA-DMB* |
| 6 | 38 | rs171329 | 33012639 | *HLA-DMB* |
| 6 | 39 | rs1480380 | 33021224 | *HLA-DMA* |
| 6 | 40 | rs3135029 | 33029752 | *HLA-DMA* |
| 6 | 41 | rs9378127 | 33030437 | *HLA-DMA* |
| 6 | 42 | rs209474 | 33032562 | *HLA-DMA* |
| 6 | 43 | rs176248 | 33073920 | *HLA-DOA* |
| 6 | 44 | rs206762 | 33078428 | *HLA-DOA* |
| 6 | 45 | rs3128931 | 33079686 | *HLA-DOA* |
| 6 | 46 | rs3129304 | 33081721 | *HLA-DOA* |
| 6 | 47 | rs86567 | 33084737 | *HLA-DOA* |
| 6 | 48 | rs403414 | 33085293 | *HLA-DOA* |
| 6 | 49 | rs3763342 | 33086975 | *HLA-DOA* |

| | | | | |
|---|---|---|---|---|
| 6 | 50 | rs9276991 | 33089826 | *HLA-DOA* |
| 6 | 51 | rs3130604 | 33093030 | *HLA-DOA* |
| 6 | 52 | rs6933994 | 33095098 | *HLA-DOA* |
| 6 | 53 | rs9296068 | 33096673 | |
| 6 | 54 | rs763469 | 33112365 | |
| 6 | 55 | rs2395309 | 33134224 | *HLA-DPA1* |
| 6 | 56 | rs3077 | 33141000 | *HLA-DPA1* |
| 6 | 57 | rs2301226 | 33142574 | *HLA-DPA1* |
| 6 | 58 | rs1126534 | 33145617 | *HLA-DPA1* |
| 6 | 59 | rs1431399 | 33149012 | *HLA-DPA1* |
| 6 | 60 | rs987870 | 33150858 | *HLA-DPA1* |
| 6 | 61 | rs9277565 | 33164875 | *HLA-DPB1* |
| 6 | 62 | rs2281390 | 33167647 | *HLA-DPB1* |
| 6 | 63 | rs2281388 | 33168096 | *HLA-DPB1* |
| 6 | 64 | rs3117222 | 33168927 | *HLA-DPB1* |
| 6 | 65 | rs3128918 | 33169076 | *HLA-DPB1* |
| 6 | 66 | rs3130192 | 33169908 | *HLA-DPB1* |

[a]The marker number assigned to each SNP corresponds to the numbers in the LD plot in Figure 1.
